# Mathematical model of uptake and metabolism of arsenic(III) in human hepatocytes - Incorporation of cellular antioxidant response and threshold-dependent behavior

**DOI:** 10.1186/1752-0509-5-16

**Published:** 2011-01-25

**Authors:** Spyros K Stamatelos, Christopher J Brinkerhoff, Sastry S Isukapalli, Panos G Georgopoulos

**Affiliations:** 1 Environmental and Occupational Health Sciences Institute (EOHSI) a joint institute of UMDNJ-Robert Wood Johnson Medical School and Rutgers University 170 Frelinghuysen Rd, Piscataway, NJ 08854 USA; 2 Department of Biomedical Engineering, Rutgers University 599 Taylor Road, Piscataway, NJ 08854 USA

## Abstract

**Background:**

Arsenic is an environmental pollutant, potent human toxicant, and oxidative stress agent with a multiplicity of health effects associated with both acute and chronic exposures. A semi-mechanistic cellular-level toxicokinetic (TK) model was developed in order to describe the uptake, biotransformation and clearance of arsenical species in human hepatocytes. Notable features of this model are the incorporation of arsenic-glutathione complex formation and a "switch-like" formulation to describe the antioxidant response of hepatocytes to arsenic exposure.

**Results:**

The cellular-level TK model applies mass action kinetics in order to predict the concentrations of trivalent and pentavalent arsenicals in hepatocytes. The model simulates uptake of arsenite (iAs^III^) via aquaporin isozymes 9 (AQP9s), glutathione (GSH) conjugation, methylation by arsenic methyltransferase (AS3MT), efflux through multidrug resistant proteins (MRPs) and the induced antioxidant response via thioredoxin reductase (TR) activity. The model was parameterized by optimization of model estimates for arsenite (iAs^III^), monomethylated (MMA) and dimethylated (DMA) arsenicals concentrations with time-course experimental data in human hepatocytes for a time span of 48 hours, and dose-response data at 24 hours for a range of arsenite concentrations from 0.1 to 10 μM. Global sensitivity analysis of the model showed that at low doses the transport parameters had a dominant role, whereas at higher doses the biotransformation parameters were the most significant. A parametric comparison of the TK model with an analogous model developed for rat hepatocytes from the literature demonstrated that the biotransformation of arsenite (e.g. GSH conjugation) has a large role in explaining the variation in methylation between rats and humans.

**Conclusions:**

The cellular-level TK model captures the temporal modes of arsenical accumulation in human hepatocytes. It highlighted the key biological processes that influence arsenic metabolism by explicitly modelling the metabolic network of GSH-adducts formation. The parametric comparison with the TK model developed for rats suggests that the variability in GSH conjugation could have an important role in inter-species variability of arsenical methylation. The TK model can be incorporated into larger-scale physiologically based toxicokinetic (PBTK) models of arsenic for improving the estimates of PBTK model parameters.

## Background

Arsenic is a naturally occurring metalloid, abundant in the earth's crust and a component of more than 245 minerals [1]. Exposure to arsenic has been associated with cancers of the liver, bladder, skin and lung [2,3]. Epidemiological studies in Taiwan, Bangladesh and India have reported adverse health effects associated with chronic arsenic exposure including; chronic obstructive pulmonary disease, non-cirrhotic portal fibrosis, hypertension and ischeamic heart disease [4]. The risk of developing serious diseases from chronic exposure to inorganic arsenic in drinking water prompted the US Environmental Protection Agency (EPA) to lower the maximum contamination level (MCL) for arsenic in drinking water to 10 ppb [5].

There are two biologically important arsenic valence states: arsenite (As(OH)^3^, iAs^III^) and arsenate (AsO(OH)^3^, iAs^V^). Inorganic arsenic in water is largely in the form of arsenate; it is negatively charged at physiological pH and slowly taken up by cells [6]. Arsenate is rapidly converted to arsenite *in vivo *[7] which is taken up by cells much more quickly than arsenate [8]. Methylation of arsenicals facilitates their excretion from the cell and therefore was long considered a detoxification process, but recent evidence indicates that monomethylated (MMA) and dimethylated (DMA) arsenicals have many toxic effects including increased oxidative stress [9], chromosomal aberrations (CA), and oxidative DNA damage [10-12]. In hepatocytes, trivalent monomethylated arsenicals (MMA^III^) inhibit the activity of thioredoxin reductase (TR), which is a critical antioxidant enzyme controlling the cellular redox balance [13,14].

Uptake and efflux of arsenicals occur primarily through transporter proteins. Uptake of iAs^III ^in hepatocytes and efflux of MMA^III ^to blood take place through aquaporin isozymes 9 (AQP9), a family of membrane-spanning proteins that facilitate movement of solutes down their concentration gradient. AQP9 channels are expressed at high concentrations in liver cells and have been shown to transport iAs^III ^when expressed both in *Saccharomyces cerevisiae *(yeast) and in *Xenopus oocytes *[15-17]. Another class of transmembrane proteins facilitating the transport of iAs^III ^and MMA^III ^across the cellular membrane of hepatocytes is glucose transporters and especially GLUT2 which is highly expressed in the liver [18,19]. Glutathione conjugated arsenicals are exported to the extracellular space via multidrug resistant proteins (MRPs) and multidrug resistant P-glycoproteins (PGPs)which are ATP-binding cassette (ABC) transporters that export solutes against their concentration gradient [19-21].

Methylation of inorganic arsenic takes place primarily in the liver and specifically in hepatocytes via enzymatic catalysis by arsenic methyltransferase (AS3MT), previously known as Cyt19, producing both mono- and dimethylated arsenicals [22-25]. Two biochemical pathways have been proposed for methylation of arsenates with a key difference in the substrate for AS3MT methylation: (a) a classical process of alternating steps of reduction and oxidative methylation where iAs^III ^and MMA^III ^are the substrates and the methylation can happen in the presence or absence of GSH [26] and (b) a process of GSH conjugation and reductive methylation where thiol-bound arsenicals (arsenic triglutathione - ATG, monomethylarsenic diglutathione - MADG) are the substrates [27,28]. GSH has a stimulatory role in both methylation pathways either as a reductant or in direct conjugation with arsenicals [29].

Arsenic activates the redox sensitive transcription factor Nuclear Factor -E2- related factor 2 (Nrf2) causing its increased nuclear translocation and Nrf2 binding to the Antioxidant Response Element (ARE) [30,31]. Arsenic activates Nrf2 in a different manner when compared to other compounds such as sulforaphane (SF) and tert-butylhydroquinone (tBHQ) enhancing the interaction of specific subunits of the E3 ubiquitin ligase [32]. It has been suggested that hepatocytes exhibit an "on-switch" antioxidant behavior when exposed to increasing arsenic doses [33], possibly a result of this Nrf2 activation.

**Figure 1:**
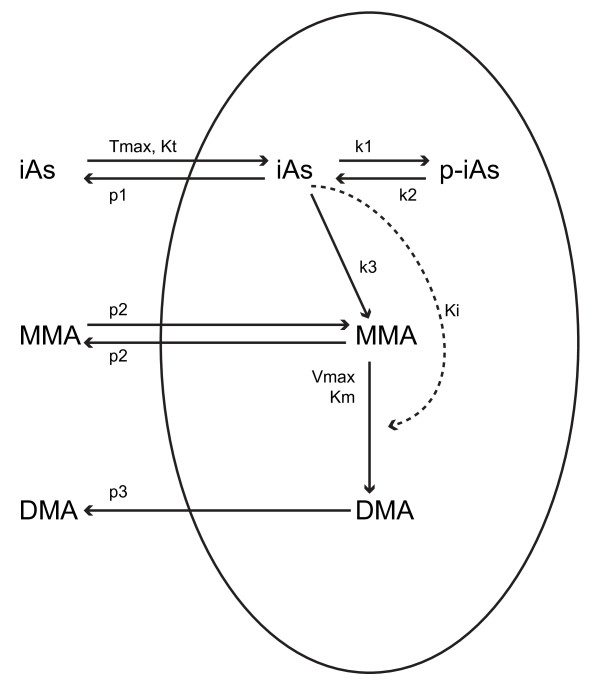
**Schematic depiction of the cellular level TK model for rat hepatocytes**. The solid lines that cross the cellular membrane (oval) represent transport processes, while the solid lines within the oval represent biotransformation. The dashed line represents the inhibitory effect of iAs^III ^on the second methylation reaction (MMA to DMA) (Source: Easterling *et al. *[34])

In this study, a cellular-level semi-mechanistic TK model was developed for predicting intra-cellular concentrations of different arsenicals (trivalent and pentavalent) in hepatocytes. Currently, the only published cellular-level TK model for the uptake, biotransformation and efflux of arsenicals is the Easterling *et al. *[34] model schematically shown in Figure 1; they demonstrated the relative importance of transport processes affecting the accumulation of arsenicals in rat hepatocytes [34]. However, a TK model for humans is needed since the inherent variation of arsenic metabolic capacity across various organisms complicates the extrapolation of rat TK model to humans. Therefore, the mathematical model presented here was developed for humans and parameterized based on data from human hepatocytes.

## Methods

The human TK model applies mass action kinetics in order to predict the concentrations of trivalent and pentavalent arsenicals including arsenite (iAs^III^), monomethylated (MMA), and dimethylated arsenicals (DMA) in human hepatocytes. This TK model takes into account processes such as influx, efflux, methylation, oxidation and glutathione conjugation of arsenicals. Moreover, it accounts for induced cellular antioxidant response due to arsenic exposure through a "switch-like" mechanism that alters the model response above a specific threshold concentration [35].

This model has been compared with the Easterling *et al. *model [34] , in terms of the ability to fit to data of arsenic retention and methylation in human hepatocytes. This comparison aims to highlight the advantages of developing biologically relevant TK models based on data acquired from human cells. Further comparison of these two models in terms of their estimated parameter values aim to study the major intracellular kinetic processes that contribute to the differences in metabolism between humans and rats.

### Model Development

**Figure 2:**
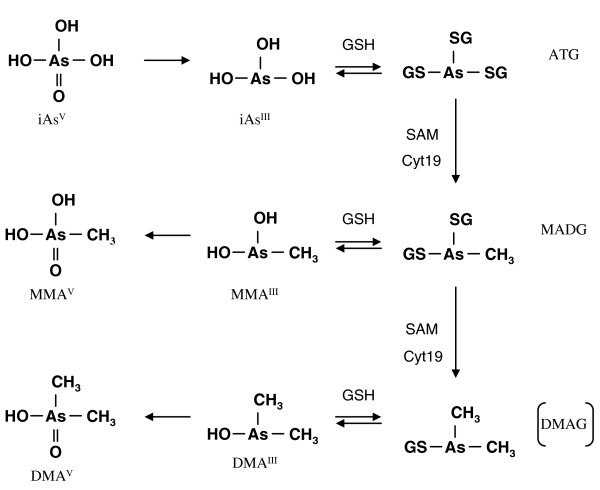
**A new metabolic pathway of inorganic arsenic biotransformation via arsenic-GSH complexes formation**. This pathway includes two separate branches of arsenic biotransformation: MADG → MMA^III ^→ MMA^V ^and MADG → DMAG → DMA^III ^→ DMA^V ^(Source: Hayakaya *et al. *[27])

**Figure 3:**
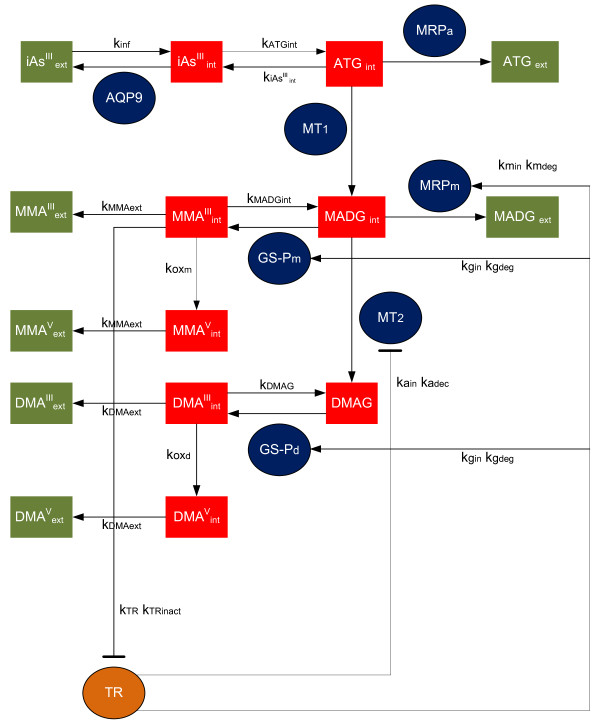
**Schematic depiction of the cellular level TK model for human hepatocytes showing the major components**. Green squares represent extracellular amounts of arsenicals, while red squares represent intracellular amounts. Ovals represent activities of proteins (AQP9, TR, MT1, MT2, MRPa and MRm) and GSH (GS-Pm, GS-Pd). Arrows and hammerheads indicate activation and inhibition respectively.

The semi-mechanistic TK model describes arsenic transport across the cellular membrane and arsenic metabolism in hepatocytes according to the metabolic reaction cascade proposed by Hayakawa *et al. *[27] (Figure 2). Figure 3 presents a schematic depiction of the constituents of the TK model that are also explained in Table 1. These constituents include chemical species and enzymes, and the interactions among them. Fundamental assumptions made in the formulation of the TK model are:

**Table 1:** Optimized parameter values of the TK model along with the corresponding process they describe

Notation	Parameter	Value	Units	Description
1	k_ATG int_	0.25	1/min	Rate of production of ATG

2	k_MADG int_	90	1/min	Rate of production of MADG

3	k_DMAG_	0.0122	1/min	Rate of production of DMAG

4	k_oxd_	33.254	1/min	Rate of oxidation of DMA^III^

5	k_oxm_	0.2375	1/min	Rate of oxidation of MMA^III^

6	k_iAs_^III^_int_	320	1/min	Rate of production of iAs^III^

7	k_MMA_^III^_int_	1200	1/min	Rate of production of MMA^III^

8	n_3_	8	-	Hill coefficient of inhibition of MMA^III ^production

9	Kd_3_	12.94	μM	Dissociation constant of inhibition of MMA^III^

10	f_GSHm_	0.992	-	Coefficient of inhibition of MADG hydrolysis

11	kg_in_	704.96	1/min	Rate of GSH production increase

12	k_DMA_^III^_int_	0.8472	1/min	Rate of production of DMA^III^

13	f_GSHd_	0.9988	-	Coefficient of inhibition of DMAG hydrolysis

14	Vmax_t2_	1.237	pmol/min	Maximal rate of MADG efflux

15	Km_t2_	19.47	μM	Half saturation constant of MADG efflux

16	km_in_	512.27	1/min	Rate of upregulation of MRP

17	k_ss_	4.26	1/min	Steady state rate of efflux of iAs^III^

18	τ_e_	10	min	Time constant of AQP9 inactivation

19	k_0_	4.2	1/min	Initial rate of efflux of iAs^III^

20	k_inf_	1.613	1/min	Influx of iAs^III^

21	k_MMAext_	0.0006	1/min	Rate of efflux of MMA

22	f_m_	0.2	-	Coefficient of efflux of MMA

23	Vmax_t1_	0.35	pmol/min	Maximal rate of ATG efflux

24	Km_t1_	2.3	μM	Half saturation constant of ATG efflux

25	k_DMAext_	0.002	1/min	Rate of efflux of DMA

26	f_d_	3	-	Coefficient of efflux of DMA

27	Vmax_m_	50	pmol/min	Maximal rate of ATG methylation

28	Km_m_	9.32	μM	Half saturation constant of ATG methylation

29	n_1_	1.22	-	Hill coefficient of ATG methylation

30	Kd_1_	0.315	μM	Dissociation constant of ATG methylation

31	K_im_	1.53	μM	Inhibition constant of ATG methylation

32	K_id_	1	μM	Inhibition constant of MADG methylation

33	Vmax_d_	8	pmol/min	Maximal rate of MADG methylation

34	Km_d_	0.034	μM	Half saturation constant of MADG methylation

35	n_2_	1.83	-	Hill coefficient of MADG methylation

36	Kd_2_	2.33	μM	Dissociation constant of MADG methylation

37	ka_in_	1.64	1/min	Rate of AS3MT efficiency increase

38	f_A_	50	-	Coefficient of MADG methylation inactivation

39	k_TR_	0.99	1/min	Rate of TR signaling

40	k_TRinact_	987.13	1/min	Rate of TR inactivation

41	TR_C_	15	-	Constant of TR inactivation

42	N	1.75	-	Hill coefficient of TR inactivation

43	TR_0_	9.27	1/min	Steady state value of TR activity

1) Arsenite influx across the cellular membrane pores is governed by their electrochemical potential, and can be described through an ion channel conductance-based formulation.

2) The oxidative stress mediated response of hepatocytes to arsenite exposure exhibits a "switch-like" behavior, and the upregulation of enzyme activities can be described through an approximate step function at a threshold concentration.

3) The methylation reactions are influenced by cooperativity phenomena as well as substrate inhibition, and can be described through a hybrid approach of Hill and Michaelis-Menten kinetics.

4) The GSH-bound hydrolysis and clearance of methylated arsenicals exhibit a threshold-dependent behavior, and can be described using a sigmoidal function.

5) Concentrations of arsenicals are uniform within the hepatocytes as well as the extracellular medium

6) All hepatocytes in the system have identical properties, are uniformly distributed in the medium, and are exposed to the same extracellular concentrations of arsenicals.

Uptake of arsenite by hepatocytes via AQP9s [36] is governed by their electrochemical potential across the cellular membrane (for simplicity we refer to AQP9s as being the ensemble of the activity of both AQP9 and GLUT2 channels). The conductance-based formulation for ion channels proposed in the Hodgkin-Huxley model [37] is used here to describe the regulation of arsenite flux by AQP9s (Equations 1-2). Specifically, the inactivation of AQP9 subunit gates during iAs^III ^influx is described by Equation 1b, which expresses the increased probability of these gates closing as more transport across the gates occurs.(1a)

(1b) $$\text{AQP9}={\text{[k}}_{\text{0}}+{\text{(k}}_{\text{SS}}-{\text{k}}_{\text{0}}\text{)}\times {\text{(1-e}}^{-\frac{\text{t}}{\tau_{\text{e}}}}\text{)]}$$

(2)

Where, k_inf _represents the mass transfer coefficient for influx of arsenite in hepatocytes; ${\text{k}}_{{\text{ATG}}_{\text{int}}}$ represents the rate constant for arsenite conjugation with GSH to form ATG catalyzed by the Glutathione S-Transferase (GST) family of enzymes; ${\text{k}}_{{\text{iAs}}^{\text{III}}{}_{\text{int}}}$ is the rate constant for hydrolysis of ATG (reciprocal to conjugation); k_SS _is the steady state rate constant for efflux of arsenite that is attained at long time periods; k_0 _is the rate constant describing the basal activity of AQP9; and τ_e _is the time constant governing the regulation of AQP9 gates.

Thioredoxin (Trx) Reductase (TR) is the enzyme that catalyzes the reduction of Trx. Trx is a critical antioxidant protein and an important reductant in the methylation of arsenic by AS3MT [38]. The inactivation of TR by MMA^III ^leads to signals that account for two different phenomena: the induction of GSH and ABC transporters via a redox sensitive activation of the cellular antioxidant response Nrf2 nuclear receptor pathway [14,39] and the decreased methylation capacity of AS3MT. In this study the main focus is on MRPs as an efflux mechanism of arsenicals since it has been reported that they are regulated by Nrf2 [40,41]. The inactivation is modeled using principles of indirect response model theory [42,43] via the threshold-dependent parameter:$\text{S}=\frac{{{\text{[iAs}}^{\text{III}}\text{]}}_{\text{init}}-\text{threshold}}{\text{threshold}}$. Parameter S depends on the initial exposure concentration of arsenite, (iAs^III^)_init _and a threshold concentration. The value of S is zero when arsenite doses are below the threshold concentration, and gradually increases with greater arsenite doses. The following equations describe this reaction cascade:

(3a) $$\begin{array}{l} \frac{\text{dTR}}{\text{dt}}={\text{k}}_{\text{TR}}\times {\text{(TR}}_{\text{0}}-\text{TR)}-\frac{\text{S}}{{\text{TR}}_{\text{C}}}\;\\ \;\times {\text{k}}_{{\text{TR}}_{\text{inact}}}\times {\text{H}}_{\text{TR}} \end{array}$$

(3b) $${\text{H}}_{\text{TR}}=\frac{{{\text{[MMA}}^{\text{III}}\text{]}}^{\text{N}}}{{{\text{(IC}}_{\text{TR}}\text{)}}^{\text{N}}+{{\text{[MMA}}^{\text{III}}\text{]}}^{\text{N}}}$$

(4a) $$\frac{\text{dGSH}}{\text{dt}}={\text{k}}_{{\text{g}}_{\text{in}}}\times \frac{\text{1}}{\text{TR}}-{\text{k}}_{{\text{g}}_{\text{deg}}}\times \text{GSH}$$

(4b) $${\text{k}}_{{\text{g}}_{\text{deg}}}=\frac{{\text{k}}_{{\text{g}}_{\text{in}}}}{{\text{TR}}_{\text{0}}}$$

(5a) $$\frac{\text{dMRP}}{\text{dt}}={\text{k}}_{{\text{m}}_{\text{in}}}\times \frac{\text{1}}{\text{TR}}-{\text{k}}_{{\text{m}}_{\text{deg}}}\times \text{MRP}$$

(5b) $${\text{k}}_{{\text{m}}_{\text{deg}}}=\frac{{\text{k}}_{{\text{m}}_{\text{in}}}}{{\text{TR}}_{\text{0}}}$$

(6a) $$\frac{\text{dAS3MT}}{\text{dt}}={\text{k}}_{{\text{a}}_{\text{in}}}\times \text{TR}-{\text{k}}_{{\text{a}}_{\text{dec}}}\times \text{AS3MT}$$

(6b) $${\text{k}}_{{\text{a}}_{\text{dec}}}={\text{k}}_{{\text{a}}_{\text{in}}}\times {\text{TR}}_{\text{0}}$$

where, k_TR _is the first-order rate constant controlling the activity of TR; TR_0 _is the baseline activity value of TR; ${\text{k}}_{{\text{TR}}_{\text{inact}}}$ is the first-order rate constant for TR inactivation; TR_c _is a dimensionless inactivation constant; N is the Hill coefficient for enzyme inactivation from MMA^III^; ${\text{k}}_{{\text{g}}_{\text{in}}}$, ${\text{k}}_{{\text{m}}_{\text{in}}}$ and ${\text{k}}_{{\text{a}}_{\text{in}}}$ are the rate constants governing of the activities of GSH, MRP and AS3MT, ${\text{k}}_{{\text{g}}_{\text{deg}}}$, ${\text{k}}_{{\text{m}}_{\text{deg}}}$ and ${\text{k}}_{{\text{a}}_{\text{dec}}}$ are the corresponding first-order decay constants.

Methylation reactions of arsenic in the liver have been modeled in published cellular-level and whole-body PBTK models with classical Michaelis-Menten kinetics [34,44-46]. Alternatives include cooperativity models such as the classical Hill-type formulation and the more mechanistic Monod-Wyman-Changeux (MWC) model [47]. In preliminary analyses, these formulations were unable to explain the time course patterns of arsenic retention and methylation in human hepatocytes (results not shown). Therefore, an alternative, non-classical formulation was used in the TK model. In this model, the AS3MT is assumed to exhibit cooperativity and the methylation reaction rate is assumed to exhibit hysteretic sigmoidal behavior as per Frieden [48]. In this formulation, the cooperativity is described by a Hill-type formulation for V_max _that is dependent on the total ATG present in the system. The formulation accounts for the constitutive influence of GSH in the methylation reaction cascade and its role in the increase of V_max_. Moreover this Hill-type formulation for V_max _accounts for a possible colocalization of thiol-containing proteins that interact with GSH (e.g. GSTP1), MRPs and AS3MT in hepatocytes. This colocalization would control not only the production and clearance of ATG but methylation activity as well [49,50].

Previous studies have suggested that exposure of human hepatocytes to elevated doses of iAs^III ^(0.4 - 4 μΜ) markedly reduced the production of DMAs while at the same time increased the yields of MMAs [51]. Therefore, it is assumed here that the AS3MT inactivation signal (Equation 6) affects only the second methylation reaction rate (Equation 8a).

(7a) $$\begin{array}{l} {\text{MADG}}_{\text{ui}}={\text{MT}}_{\text{1}}\;\\ \;\times \frac{{\text{[ATG]}}_{\text{int}}}{\text{1}+\frac{{\text{[ATG]}}_{\text{int}}}{{\text{Km}}_{\text{m}}}\times \text{(1}+\frac{{\text{[ATG]}}_{\text{int}}}{{\text{K}}_{\text{im}}}\text{)}} \end{array}$$

(7b) $$\begin{array}{l} {\text{MT}}_{\text{1}}=\frac{{{\text{([ATG]}}_{\text{int}}+{\text{[ATG]}}_{\text{ext}}\text{)}}^{{\text{n}}_{\text{1}}}}{{{\text{(Kd}}_{\text{1}}\text{)}}^{{\text{n}}_{\text{1}}}+{{\text{([ATG]}}_{\text{int}}+{\text{[ATG]}}_{\text{ext}}\text{)}}^{{\text{n}}_{\text{1}}}}\;\\ \;\times \frac{{\text{Vmax}}_{\text{m}}}{{\text{Km}}_{\text{m}}} \end{array}$$

(8a) $$\begin{array}{l} {\text{DMAG}}_{\text{ui}}={\text{MT}}_{\text{2}}\;\\ \;\times \frac{{\text{[MADG]}}_{\text{int}}}{\text{1}+\frac{{\text{[MADG]}}_{\text{int}}}{{\text{Km}}_{\text{d}}}\times \text{(1}+\frac{{\text{[ATG]}}_{\text{int}}}{{\text{K}}_{\text{id}}}\text{)}} \end{array}$$

(8b) $$\begin{array}{l} {\text{MT}}_{\text{2}}=\frac{\text{AS3MT}}{{\text{f}}_{\text{A}}\times \text{tanh(S)}+\text{1}}\;\\ \;\times \frac{{{\text{([ATG]}}_{\text{int}}+{\text{[ATG]}}_{\text{ext}}\text{)}}^{{\text{n}}_{\text{2}}}}{{{\text{(Kd}}_{\text{2}}\text{)}}^{{\text{n}}_{\text{2}}}+{{\text{([ATG]}}_{\text{int}}+{\text{[ATG]}}_{\text{ext}}\text{)}}^{{\text{n}}_{\text{2}}}}\times \frac{{\text{Vmax}}_{\text{d}}}{{\text{Km}}_{\text{d}}} \end{array}$$

where, MADG_ui _and DMAG_ui _are the rates of arsenic methylation for the first and second methylation reactions respectively. ${\text{V}}_{{\text{max}}_{\text{m}}}$ and ${\text{V}}_{{\text{max}}_{\text{d}}}$are the maximal rates of the first and second methylation reactions respectively; ${\text{K}}_{{\text{m}}_{\text{m}}}$ and ${\text{K}}_{{\text{m}}_{\text{d}}}$ are the half-saturation constants for the methylation reactions; K_im _and K_id _are the uncompetitive inhibition constants for the respective reactions n_1_, n_2 _are the Hill coefficients; ${\text{K}}_{{\text{d}}_{1}}$ and ${\text{K}}_{{\text{d}}_{2}}$ are the dissociation constants influencing the sigmoidal change in ${\text{V}}_{{\text{max}}_{\text{m}}}$ and ${\text{V}}_{{\text{max}}_{\text{d}}}$; and f_A _is a coefficient of the second methylation reaction inactivation.

MADG hydrolysis reaction is modeled using a "switch-like" formulation. For doses below the threshold, a Hill-type formulation is used (Equation 9b). Above the threshold concentration, the rates of hydrolysis of MADG and DMAG to MMA^III ^and DMA^III^, respectively, are assumed to be attenuated due to oxidative stress-induced GSH upregulation (Equations 9-10). On the other hand, this non-linear behavior may result in an increase or a decrease of GSH depending on the concentration of iAs^III ^and the duration of exposure. The non-linear sigmoid function tanh(S) is used here to describe this "switch-like" behavior; this formula has been previously used [52] in a neurocomputational model to describe the non-linear threshold-dependent behavior of neuronal firing rate.

(9a) $$\begin{array}{l} {\text{HD}}_{\text{m}}={\text{[1-f}}_{{\text{GSH}}_{\text{m}}}\times \text{tanh(S)] }\\ \;\times \text{(1}-{\text{H}}_{\text{GSH}}+\text{tanh(S)}\times {\text{H}}_{\text{GSH}}\text{) }\\ \;\times {\text{GS-P}}_{\text{m}}\times {\text{(MADG)}}_{\text{int}} \end{array}$$

(9b) $${\text{GS-P}}_{\text{m}}=\frac{{\text{ k}}_{{\text{MMA}}^{\text{III}}{}_{\text{int}}}}{\text{GSH}}$$

(9c) $${\text{H}}_{\text{GSH}}=\frac{{{\text{([ATG]}}_{\text{int}}+{\text{[ATG]}}_{\text{ext}}\text{)}}^{{\text{n}}_{\text{3}}}}{{{\text{(Kd}}_{\text{3}}\text{)}}^{{\text{n}}_{\text{3}}}+{{\text{([ATG]}}_{\text{int}}+{\text{[ATG]}}_{\text{ext}}\text{)}}^{{\text{n}}_{\text{3}}}}$$

(10a) $${\text{HD}}_{\text{d}}={\text{[1-f}}_{{\text{GSH}}_{\text{d}}}\times \text{tanh(S)]}\times {\text{GS-P}}_{\text{d}}\times {\text{ (DMAG)}}_{\text{int}}$$

(10b) $${\text{GS-P}}_{\text{d}}=\frac{{\text{ k}}_{{\text{DMA}}^{\text{III}}{}_{\text{int}}}}{\text{GSH}}$$

where HD_m _and HD_d _are the rates of hydrolysis of MADG and DMAG respectively. ${\text{k}}_{{\text{MMA}}^{\text{III}}{}_{\text{int}}}$ is the reaction rate constant for MADG hydrolysis (MMA^III ^production); ${\text{f}}_{{\text{GSH}}_{\text{m}}}$ is the coefficient of inhibition of MADG hydrolysis; n_3 _and ${\text{K}}_{{\text{d}}_{3}}$ are the Hill coefficient and dissociation constant, respectively, for the inhibition term; ${\text{k}}_{{\text{DMA}}^{\text{III}}{}_{\text{int}}}$ is the reaction rate constant for DMAG hydrolysis (DMA^III ^production); and ${\text{f}}_{{\text{GSH}}_{\text{d}}}$ is the coefficient of inhibition of DMAG hydrolysis.

Efflux of GSH (or protein) -bound arsenic adducts (ATG, MADG) is assumed to take place via multidrug resistant proteins (MRPs), and is described by classical Michaelis-Menten kinetics. Since MADG is a substrate in the dimethylation reaction (Equation 8a), its efflux rate is assumed to be affected by MRP levels [51].

(11a) $$\frac{{\text{d(ATG)}}_{\text{ext}}}{\text{dt}}={\text{MRP}}_{\text{a}}\times \frac{{\text{[ATG]}}_{\text{int}}}{\text{1}+\frac{{\text{[ATG]}}_{\text{int}}}{{\text{Km}}_{{\text{t}}_{1}}}}$$

(11b) $${\text{MRP}}_{\text{a}}=\frac{{\text{Vmax}}_{{\text{t}}_{1}}}{{\text{Km}}_{{\text{t}}_{1}}}$$

(12a) $$\frac{{\text{d(MADG)}}_{\text{ext}}}{\text{dt}}={\text{MRP}}_{\text{m}}\times \frac{{\text{[MADG]}}_{\text{int}}}{\text{1}+\frac{{\text{[MADG]}}_{\text{int}}}{{\text{Km}}_{{\text{t}}_{\text{2}}}}}$$

(12b) $${\text{MRP}}_{\text{m}}=\text{MRP}\times \frac{{\text{Vmax}}_{{\text{t}}_{\text{2}}}}{{\text{Km}}_{{\text{t}}_{\text{2}}}}$$

(13)(14)(15)(16)

where, ${\text{V}}_{{\text{max}}_{{\text{t}}_{\text{1}}}},{\text{K}}_{{\text{m}}_{{\text{t}}_{\text{1}}}}$ and ${\text{V}}_{{\text{max}}_{{\text{t}}_{\text{2}}}},{\text{K}}_{{\text{m}}_{{\text{t}}_{\text{2}}}}$ are the Michaelis constants of the biophysical clearance of ATG and MADG, respectively; ${\text{k}}_{{\text{MMA}}_{\text{ext}}}$ and ${\text{k}}_{{\text{DMA}}_{\text{ext}}}$ are the rate constants of MMA and DMA clearance, respectively; f_m _and f_d _are the dimensionless coefficients of clearance of the respective processes affecting the maximal efflux.

The remaining biotransformation reactions include a series of methylation, glutathione conjugation and oxidation reactions [27] (Equations 17-23).

(17) $$\begin{array}{l} \frac{{\text{d(ATG)}}_{\text{int}}}{\text{dt}}={\text{k}}_{{\text{ATG}}_{\text{int}}}\times {{\text{(iAs}}^{\text{III}}\text{)}}_{\text{int}}-{\text{k}}_{{\text{iAs}}^{\text{III}}{}_{\text{int}}}\\ \;\times {\text{(ATG)}}_{\text{int}}-{\text{MADG}}_{\text{ui}}-\frac{{\text{d(ATG)}}_{\text{ext}}}{\text{dt}} \end{array}$$

(18) $$\begin{array}{l} \frac{{\text{d(MADG)}}_{\text{int}}}{\text{dt}}={\text{MADG}}_{\text{ui}}\;\\ \;+{\text{k}}_{{\text{MADG}}_{\text{int}}}\times {{\text{(MMA}}^{\text{III}}\text{)}}_{\text{int}}\;\\ \;-{\text{DMAG}}_{\text{ui}}-{\text{HD}}_{\text{m}}-\frac{{\text{d(MADG)}}_{\text{ext}}}{\text{dt}} \end{array}$$

(19)(20)(21)(22)(23)

where ${\text{k}}_{{\text{MADG}}_{\text{int}}}$ is the rate constant of MADG production catalyzed by the Glutathione S-Transferase (GST) family of enzymes; k_oxm _is the rate constant for MMA^III ^oxidation; k_DMAG _is the rate constant for DMAG production catalyzed by the Glutathione S-Transferase (GST) family of enzymes and k_oxd _is the rate constant for DMA^III ^oxidation. It has been suggested for this biotransformation pathway that trivalent arsenicals mostly bound to thiol-containing proteins are conjugated with GSH and methylated in the presence of arsenic methyltransferase (AS3MT) [28]. Therefore, the parameters k_ATGint_, ${\text{k}}_{{\text{MADG}}_{\text{int}}}$ and k_DMAG _indirectly represent the binding of trivalent arsenicals to thiol-containing proteins in the TK model.

The TK model has been implemented in MATLAB; the system of ODEs comprising the TK model is solved numerically using the stiff solver *ode15s*. First, the model parameters corresponding to low doses (i.e. below the threshold) were estimated using time course *in vitro *measurements of arsenicals following exposures to 0.1 μM iAs^III ^, from Styblo *et al. *[51]; it was assumed that at this dose, the hepatocytes exhibit no induced antioxidant response. Subsequently, model parameters corresponding to a wider range of doses (i.e. including both low-dose and high-dose behavior) were estimated using dose-response data (for doses ranging from 0.1 - 10 μM) reported by Drobna *et al. *[53]. The Drobna dataset includes measured concentrations of iAs^III^, MMA, and DMA in primary cultured human hepatocytes after 24 h exposure to iAs^III ^(data for hepatocytes from 8 donors). For this case study, data on hepatocytes from one donor (white female, aged in the 60 s, Donor C) [53] were used, since this donor had similar characteristics to the human donor in the study by Styblo *et al. *[51] . The deterministic optimization function *fmincon *was used for parameter estimation in both cases.

### Sensitivity analysis

Sensitivity analysis provides estimates of how variation of model's output can be apportioned to different sources of variation in model parameters. This quantity is given by the formula:

(24) $$\frac{{\text{var}}_{\text{P}}\left[\text{E(Y/P)}\right]}{\text{var(Y)}}=\frac{{\text{D}}_{\text{P}}}{\text{D}}$$

where Y denotes model output and P denotes the vector of model parameters; D_P _and D illustrate the partial and total variance of the model output due to variation in model parameters according to assigned statistical distributions.

The Fourier Amplitude Sensitivity Test (FAST) decomposes the total variance of model output (D) into terms of increasing dimensionality. FAST computes the Total Sensitivity Indices (TSI), which account not only for the variance due to individual parameters (D_i_), but also estimate the variance due to interaction among parameters (D_ij, _D_ijk_, etc.) The total variance for *n *dimensions is given by

(25) $$\text{D}=\sum_{\text{i=1}}^{\text{n}}{\text{D}}_{\text{i}}+\sum_{\text{i=1}}^{\text{n}}\sum_{\text{j=i+1}}^{\text{n}}{\text{D}}_{\text{ij}}+...+{\text{D}}_{1,2,..,\text{n}}$$

The model parameters were assumed to be normally distributed with a coefficient of variation up to 10%; for some parameters, the coefficient of variation was 1%. Ten thousand (10,000) samples were generated and the normal distributions for all parameters were truncated at 1% and 99% (approximately three standard deviations from the mean value). Three model outputs were selected for the sensitivity analysis: Areas under the Curve (AUCs) of total retention of MMA, DMA and iAs^III ^in human hepatocytes. The SIMLAB modeling platform [54] was used to perform the global sensitivity analysis.

### Comparison with the TK modeling formulation for rat hepatocytes

The TK model presented here was also compared with results from a published model for rat hepatocytes, in order to assess the inter-species differences and the feasibility of direct, cross-species extrapolation. Specifically, the comparison focused on major intracellular processes that influence the different metabolizing rates between these two species. First, the TK modeling formulation of Easterling *et al. *[34] was parameterized using data from Styblo *et al. *[51]. Although, a direct comparison is not possible because of major differences in model structures, a subset of parameters was selected for comparison that describe three major biochemical processes which account for similar cellular phenomena in both models. These processes include transport of arsenite across cellular membrane, methylation of arsenic, and biotransformation of AS3MT substrate (iAs in the rat model, and ATG in human TK model). Specific parameters used in comparison include (a) normalized activity of AQP9 (NP), which is defined as the ratio of influx and efflux of arsenite, (b) normalized activity of AS3MT (NM), which is defined as the ratio of the corresponding reaction parameters, and (c) bioavailability of AS3MT substrate (BMRS), which is defined as the ratio of rates of hydrolysis and conjugation of iAs for the human model, and the dissociation constant for the protein binding of arsenic in the rat model. These parameters are specified by Equation 26, and are described in Table 2. Parameters governing the efflux of arsenic were not compared because there is no direct correspondence between the modeling formulations.

**Table 2:** Comparison of selected parameter values between two hepatocyte-level TK modeling formulations

Kinetic Process	Human-Hepatocyte TK model	Rat-Hepatocyte TK model
NP - iAs^III^	0.38	0.38

NM - 1^st ^Reaction (1/min)	3.35	0.02

NM - 2^nd ^Reaction (1/min)	147.06	0.17

Biotransformation - 1^st ^MRS	0.0008	33.33

The two formulations are developed based on data of arsenic uptake and biotransformation in human and rat hepatocytes (NP: Normalized AQP9 activity, NM: Normalized AS3MT activity, MRS: Methylation Reaction Substrate)

In order to facilitate direct comparison, approximate volumes of the cell cultures used in different experiments (per well) were estimated. Hepatocytes were assumed to have a spherical shape with 25 μm diameter for both humans and rats [55]. Cellular volumes (Volc) for human and rat were estimated to be 1.6 and 0.8 μL respectively, based on the number of cells used in human hepatocytes experiments (2*10^5^) and rat hepatocytes experiments (10^5^) [34,51].

(26) $$\text{Human TK}:\left\{ \begin{array}{l} \text{NP}=\frac{{\text{k}}_{\text{inf}}}{{\text{k}}_{\text{0}}}\\ {\text{NM (1}}^{\text{st}}\text{)}=\frac{{\text{Vmax}}_{\text{m}}}{{\text{Km}}_{\text{m}}\times \text{Volc}}\;\\ {\text{NM (2}}^{\text{nd}}\text{)}=\frac{{\text{Vmax}}_{\text{d}}}{{\text{Km}}_{\text{d}}\times \text{Volc}}\\ \text{BMRS}=\frac{{\text{k}}_{{\text{ATG}}_{\text{int}}}}{{\text{k}}_{{\text{iAs}}^{\text{III}}{}_{\text{int}}}} \end{array}\right.$$

$$\text{Rat TK:}\left\{ \begin{array}{l} \text{NP}=\frac{{\text{T}}_{\text{max}}}{{\text{K}}_{\text{t}}\times \text{Volc}\times {\text{p}}_{\text{1}}}\\ {\text{NM (1}}^{\text{st}}\text{)}={\text{k}}_{\text{3}}\\ {\text{NM (2}}^{\text{nd}}\text{)}=\frac{\text{Vmax}}{{\text{K}}_{\text{m}}\times \text{Volc}}\\ \text{BMRS}=\frac{{\text{k}}_{2}}{{\text{k}}_{1}} \end{array}\right.$$

## Results and Discussion

**Figure 4:**
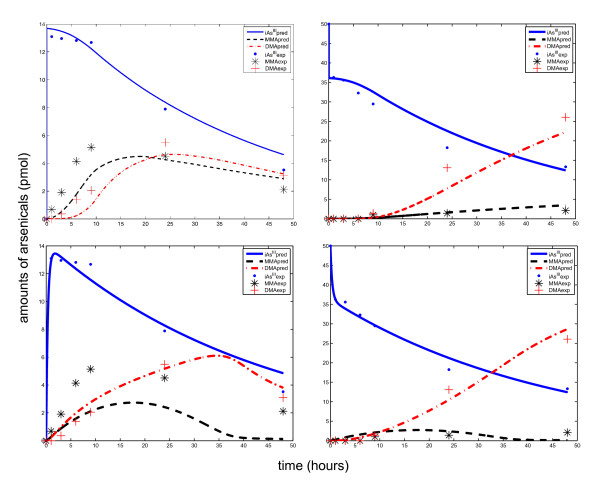
**Predicted time course profiles of arsenicals in human hepatocytes using two TK model formulations**. The top row shows estimates from the TK model presented here, while the bottom row shows estimates from the TK model formulated using the Easterling *et al. *[34] approach. The left column shows intracellular levels of iAs^III^, total MMA, and total DMA, while the right column shows corresponding extracellular levels. Experimental data are from Styblo *et al. *[51] (exposures to 0.1 μM arsenic for 2 days)

The semi-mechanistic TK model was parameterized using the *fmincon *function in MATLAB and time course data of arsenicals in human hepatocytes from Styblo et al. [51]. The parameters are shown in Table 1. The model was able to capture the three distinct modes of the time course patterns corresponding to experimental data (shown in Figure 4, Row 1). In contrast, the only currently existing cellular level TK model for arsenic, from Easterling *et al. *[34], was parameterized using the same optimization technique and data, but the model was not able to adequately capture these modes (shown in Figure 4, Row 2).

The time-course estimates from the TK model show that initially (within first minutes of exposure) the rate of influx of AQP9s is substantially higher than the metabolism, thus leading to a fast accumulation of arsenite inside the cells. Then, the influx is reduced, and metabolism increases, thus leading to a slow decrease in arsenite levels (till 8 to 9 hours). During this period, MMA production appears to be the dominant process, as shown by higher levels of MMA compared to DMA, attributable to the high rate of MADG hydrolysis. Subsequently, the arsenite concentrations decrease at a faster rate, the second methylation reaction becomes more significant, and MADG hydrolysis is inhibited (Equation 9).

**Figure 5:**
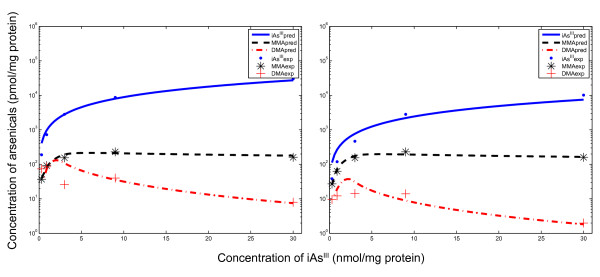
**Predicted dose-response profiles in human hepatocytes using the cellular level human TK model**. The left panel shows total amounts (in hepatocytes and the medium) of iAs^III^, MMA and DMA. The right panel shows corresponding intracellular levels. Experimental data are from Drobna *et al. *[53] for hepatocytes from a 63 year old white female

**Figure 6:**
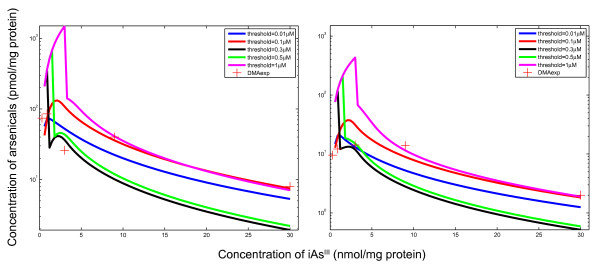
**Sensitivity testing of TK model predictions for varying assumptions of threshold levels**. The left panel shows total amounts (in hepatocytes and the medium) of DMA, while the right panel shows intracellular levels of DMA. Experimental data are from Drobna *et al. *[53] for hepatocytes from a 63 year old white female

Figure 5 shows the dose-response profiles estimated by the TK model parameterized using Drobna *et al. *data [53]. The model explained the dose-response profiles in the data, and captured the significant decrease in DMA amounts at higher arsenite doses. Based on the sensitivity testing the threshold concentration value of 0.1 μΜ was able to adequately explain the arsenicals retention and metabolism, as shown in Figure 6. Threshold values above 0.1 μΜ overestimate the concentration of DMA in hepatocytes by one order of magnitude in the low dose region. On the other hand, threshold values below 0.1 μΜ (e.g. 0.01 μΜ) underestimate the DMA concentration substantially.

**Figure 7:**
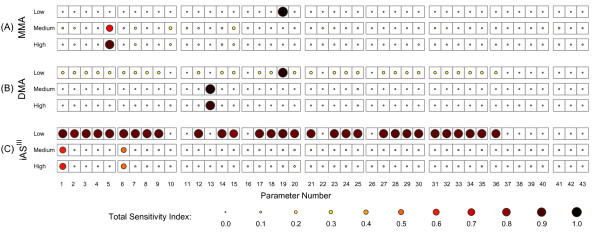
**Sensitivity analysis of intracellular MMA, DMA and iAs^III ^estimates**. Total Sensitivity Indices (TSI) of TK model parameters with respect to intracellular MMA (A), DMA (B) and iAs^III ^(C) levels for three dose scenarios: 0.1 μM - top, 1 μM - bottom left, 10 μΜ - bottom right

Results of the sensitivity analysis showed that the relative contribution of variance of individual TK model parameters varied significantly across different doses of arsenite. As shown in Figure 7A, at low doses (0.1 μΜ), the transport parameter k_0 _(Parameter 19) contributes most to the variance in intracellular MMA levels. This agrees with Easterling *et al. *[34], who reported that the transport parameters are the most significant in relation to intracellular concentration of arsenicals. On the other hand, at higher doses, the parameters related to intracellular biotransformation of MMA are the most influential. For 1 μM dose of iAs^III ^the most significant parameters are k_oxm _(Parameter 5), ${\text{f}}_{{\text{GSH}}_{\text{m}}}$ (Parameter 10), ${\text{k}}_{{\text{MMA}}^{\text{III}}{}_{\text{int}}}$ (Parameter 7) and ${\text{K}}_{{\text{m}}_{{\text{t}}_{\text{2}}}}$ (Parameter 15). The first three parameters (Parameters 5, 10, and 7) directly influence oxidation and glutathione conjugation reactions involving MMA^III^, whereas the Michaelis constant (Parameter 15) controls the activity of MRPs that efflux MADG from the cells. At high doses, induced antioxidant response of hepatocytes to arsenic leads to increased production of GSH and MRPs in the cells, leading to higher production of MADG, which can be readily effluxed via membrane-associated proteins. On the other hand, when the oxidation reaction is dominant (k_oxm_; Parameter 5), it results in higher production of MMA^V^, which becomes accumulated in the cells, leading to overall increase in intracellular MMA levels.

Figure 7B shows sensitivity analysis results for intracellular DMA levels. Similar to results from sensitivity analysis of MMA levels (Figure 7A), the transport parameter k_0 _(Parameter 19) is the most influential parameter at low doses (0.1 μΜ). For 1 μM dose, DMA production is significantly influenced primarily by ${\text{f}}_{{\text{GSH}}_{\text{d}}}$ (Parameter 13), which affects the rate of DMAG hydrolysis. Furthermore, the oxidation reaction is not important in the case of DMA levels, because DMA transport across the cellular membrane is much faster compared to MMA transport [56]. This is also corroborated by the relative values of corresponding transport parameters for MMA and DMA (f_d _>> f_m,_, as shown in Table 1), and by the findings of Styblo *et al. *[51].

Figure 7C shows sensitivity analysis results for intracellular iAs^III ^levels. At low doses (0.1 μΜ), the TSIs of most parameters are close to 1, indicating very high contributions. This unusual finding can be attributed to large interaction effects among multiple model parameters on the model output (i.e. binary interactions terms such as D_ij,,_and tertiary interaction terms such as D_ijk_). This was verified by computing first-order sensitivity indices, which account for contribution of each individual parameter (D_i_) to the output variance without taking into account higher-order interactions [57,58]; these indices for all parameters were low (< 0.1) at low doses (results not shown). At higher doses, ${\text{k}}_{{\text{ATG}}_{\text{int}}}$ (Parameter 1) and ${\text{k}}_{{\text{iAs}}^{\text{III}}{}_{\text{int}}}$ (Parameter 6) are the most influential. Both these parameters correspond to rate constants in the bidirectional reaction of ATG with iAs^III ^(glutathione conjugation of arsenite and ATG hydrolysis).

A fundamental hypothesis in this modeling formulation that allows the capturing of the dose-response profiles of arsenic retention and methylation across doses (Figure 5), is the introduction of threshold-dependent non-linear ("switch-like") mechanisms in the metabolic network due to oxidative stress (TR inactivation). This assumption is based on findings that signaling motifs exhibit biological switches under a narrow range of endogenous or exogenous stimuli [59]. This is often described by a Hill equation with a large Hill coefficient (e.g., kinase cascades [60] and nuclear-receptor pathways [61,62]). The large Hill coefficient for inhibition of MADG hydrolysis (Table 1 - Parameter 8) points to potential "switch-like" behavior of the activation of Nrf2 due to arsenic-mediated oxidative stress [32].

The parametric comparison between human and rat hepatocyte TK models for arsenic, presented in Table 2, provides insight into factors that affect arsenic metabolism in hepatocytes. Specifically, the AS3MT activity is found to be not a significant factor. Rats have been reported to be much faster metabolizers of arsenic than humans [51], but based on this study this is attributed to other factors. Specifically, in the rat-hepatocyte TK model (Figure 1), the protein-bound arsenite (p-iAs) is biotransformed to iAs (substrate of methylation reaction) at a much higher rate (five orders of magnitude) compared to the biotransformation of iAs^III ^to ATG (substrate of methylation reaction) of the human-hepatocyte TK model (Figure 3). This variability could be influenced by polymorphisms related to GSH production in hepatocytes [63] or availability of thiol-containing proteins to interact with AS3MT [28].

This work demonstrates the development of a prototype semi-mechanistic toxicokinetic (TK) model for arsenicals in human hepatocytes introducing features such as cooperativity and "switch-like" antioxidant response. Even though this model is not directly applicable to *in vivo *systems as a standalone formulation, it can be applied to inform macroscopic metabolism-related parameters in the PBTK model. On the other hand, more experimental studies on arsenicals in human hepatocytes will substantially improve model structure and can help in characterizing inter-individual variability in arsenic metabolism. Currently, the Styblo *et al. *study [51] is the only study in the authors' knowledge that reports time course profiles of arsenic methylation in human hepatocytes. Furthermore, significant uncertainties exist in experimental data due to the limitations of widely used techniques such as hydride generation-atomic absorption spectroscopy (HG-AAS) and high performance liquid chromatography-inductively coupled plasma-mass spectrometry (HPLC-ICP-MS), where glutathione conjugated arsenic species ATG and MADG have been reported to be degraded to iAs^III ^and MMA^III ^during the speciation analysis in the bile of rats exposed to arsenic [64,65].

This cellular-level TK model is based on an arsenic biotransformation pathway where arsenic-GSH adducts (ATG, MADG) are substrates for the respective methylation reactions [27,28]. It should be pointed out that arsenic can be efficiently methylated even in the absence of GSH [49,66], indicating that arsenic-GSH complexes need not be major species in the methylation of arsenic. On the other hand, the explicit consideration of arsenic-GSH complexes allows the description of a hysteresis behavior associated with methylation reactions and the stimulating role of GSH in these processes (Equations 7-8); so, this mechanism has been selected in this model. It should be noted that it is beyond the scope of this manuscript to comparatively evaluate the different arsenic biotransformation mechanisms.

Clearly it is, in principle, possible to incorporate into this cellular level TK model both the oxidative and reductive mechanisms as individual pathways. However, currently available experimental data are not adequate for estimating the relative contributions of each pathway. Development of improved experimental techniques for quantifying binding of arsenicals to GSH and thiol-containing proteins will allow the estimation of the relative contribution of each pathway. Since AS3MT coexists in hepatocytes with a number of competing elements that affect its action, its activity should be determined based on the availability of each of these elements. For instance, to study the effectiveness of the oxidative mechanism, it is possible to knock out GSH biosynthesis in hepatocytes by interfering with the activity of Glutamate-Cysteine Ligase (GCL) [67], and exposing them to various doses of arsenite. Such data sets can be used to estimate AS3MT activities along the two competing reaction pathways; this type of information is necessary in order to extend the mathematical formulation of the model described here to include both competing methylation pathways.

Parameter identification is an important issue in computational biology since most of the models involve more parameters than the available data. The TK model was parameterized using data on total arsenic of three species (iAs, MMA, DMA) and it was able to capture the modes of arsenic retention and methylation in human hepatocytes, but was not able to exactly capture the time-course profiles from the experimental data. In order to reduce the uncertainty associated with this issue, sensitivity analysis and testing were conducted as a means to identify the relative impact of each parameter on model predictions [68]. Additional time-course data (either on intermediate species or under more exposure/dose conditions) can improve model performance, and can help obtain additional mechanistic insights into the dynamics of arsenicals in hepatocytes.

## Conclusions

A cellular-level TK model was developed based on a recently proposed pathway of arsenic biotransformation. This model can describe uptake, retention and clearance of arsenicals in human hepatocytes using a semi-mechanistic approach. It highlights the key biological processes that influence arsenic metabolism by explicitly modelling the metabolic network of GSH-adducts formation [27]. Moreover, comparison of the model structure and parameters with a rat-hepatocyte TK model [34] highlights the relative roles of different metabolic reactions in the methylation of arsenic. Ongoing work involves incorporating this cellular-level semi-mechanistic TK model as a module within a whole-body PBTK model of arsenic [44], in order to improve the PBTK model parameterization and its predictions [69].

## Authors' contributions

SKS conceived the study as part of his doctoral dissertation, developed and implemented the mathematical model, analyzed the results and drafted the manuscript. CJB and SSI contributed to the development of the mathematical model, analysis of the results and manuscript drafting. PGG conceived the study and supervised the work. All authors read and approved the manuscript.
